# Hoarseness and vocal tract discomfort and associated risk factors in air traffic controllers^☆^

**DOI:** 10.1016/j.bjorl.2018.02.009

**Published:** 2018-04-05

**Authors:** Gustavo Polacow Korn, Anna Carolina Villar, Renata Rangel Azevedo

**Affiliations:** Universidade Federal de São Paulo (UNIFESP), Departamento de Otorrinolaringologia e Cirurgia de Cabeça e Pescoço, São Paulo, SP, Brazil

**Keywords:** Risk factors, Hoarseness, Workplace, Voice, Fatores de risco, Rouquidão, Local de trabalho, Voz

## Abstract

**Introduction:**

An air traffic controller is a professional who performs air traffic control functions in air traffic control units and is responsible for controlling the various stages of a flight.

**Objective:**

To compare hoarseness and vocal tract discomfort and their risk factors among air traffic controllers in the approach control of São Paulo.

**Methods:**

In a cross-sectional survey, a voice self-evaluation adapted from to self-evaluation prepared by the Brazilian Ministry of Labor for teachers was administered to 76 air traffic controllers at approach control of São Paulo, Brazil.

**Results:**

The percentage of hoarseness and vocal tract discomfort was 19.7% and 38.2%, respectively. In relation to air pollution, the percentages of hoarseness and vocal tract discomfort were higher among those who consider their working environment to be intolerable than among those in a comfortable or disturbing environment. The percentage of hoarseness was higher among those who seek medical advice due to vocal complaints and among those who experience difficulty using their voice at work than among those who experience mild or no difficulty. The percentage of vocal tract discomfort was higher among those in a very tense and stressful environment than among those who consider their work environment to be mild or moderately tense and stressful. The percentage of vocal tract discomfort was higher among those who describe themselves as very tense and stressed or tense and stressed than among those who describe themselves as calm. Additionally, the percentage of vocal tract discomfort was higher among those who care about their health.

**Conclusion:**

Among air traffic controllers, the percentage of vocal tract discomfort was almost twice that of hoarseness. Both symptoms are prevalent among air traffic controllers who considered their workplace intolerable in terms of air pollution. Vocal tract discomfort was related to a tense and stressful environment, and hoarseness was related to difficulty using the voice at work.

## Resumo

**Introdução:**

O controlador de tráfego aéreo é um profissional que executa funções de controle de tráfego aéreo em unidades de controle de tráfego aéreo e são responsáveis por controlar as várias fases de um voo.

**Objetivo:**

Comparar a rouquidão e o desconforto no trato vocal e seus fatores de risco em controladores de tráfego aéreo no centro de controle de aproximação de São Paulo.

**Método:**

Em um estudo transversal, uma autoavaliação de voz adaptada da autoavaliação preparada pelo Ministério do Trabalho para professores foi administrada a 76 profissionais do centro de controle de aproximação de São Paulo, Brasil.

**Resultados:**

A porcentagem de rouquidão e desconforto no trato vocal foi de 19,7% e 38,2%, respectivamente. Em relação à poluição do ar, as porcentagens de rouquidão e desconforto no trato vocal foram maiores entre aqueles que consideram seu ambiente de trabalho como intolerável do que entre aqueles em um ambiente confortável ou incômodo. A porcentagem de rouquidão foi maior entre aqueles que procuram assistência médica devido a queixas vocais e entre aqueles que têm dificuldade de usar a voz no trabalho do que entre aqueles que experimentam dificuldade leve ou não apresentam dificuldades. A porcentagem de desconforto no trato vocal foi maior entre aqueles em um ambiente muito tenso e estressante do que entre aqueles que consideram seu ambiente de trabalho leve ou moderadamente tenso e estressante. A porcentagem de desconforto no trato vocal foi maior entre aqueles que se descrevem como muito tensos e estressados ou tensos e estressados do que entre aqueles que se descrevem como calmos. Além disso, a porcentagem de desconforto no trato vocal foi maior entre aqueles que se preocupam com sua saúde.

**Conclusão:**

Entre os controladores de tráfego aéreo, a porcentagem de desconforto no trato vocal foi quase o dobro da rouquidão. Ambos os sintomas são prevalentes entre os controladores de tráfego aéreo que consideram o seu local de trabalho intolerável em termos de poluição do ar. O desconforto no trato vocal foi associado a um ambiente tenso e estressante, e a rouquidão foi associada à dificuldade de usar a voz no trabalho.

## Introduction

Professional voice use is defined as oral communication by people who depend on their voice to perform their working activities.^1^ Approximately one-third of current professions use the voice to varying degrees.^2^ Among professionals who rely on their voices, teachers are the focus of most studies on professional voice use.^3^, ^4^, ^5^, ^6^, ^7^, ^8^, ^9^, ^10^

As another professional voice user, Air Traffic Controllers (ATCs) have been the focus of few studies on voice problems and communicative competence.^11^

An ATC is a professional who performs air traffic control functions in air traffic control units under the Brazilian Aeronautics Command. These professionals are responsible for controlling the various stages of a flight.^12^

An aircraft normally passes through three levels of traffic control between takeoff and landing. The tower is responsible for the aircraft until it loses visual contact with the aircraft, while the Approach Control (APP) and the Area Control Center (ACC) are both responsible for the direction of the plane in the air.^11^

The function of APP controllers is to ensure a minimum distance between planes under their command near the airport and to indicate by radio the coordinates (headings), speeds, and altitudes that the pilot must adopt to fly with maximum safety and avoid collisions.^12^ This is a highly dynamic task that requires the special attention of these controllers because in addition to providing the final sequence for landing, ATCs must separate the aircraft that are landing from those that are taking off. Their voice is therefore of the utmost importance, and any vocal alterations that influence the transmission of their voice to pilots, even if temporary, may put hundreds of lives at risk.^11^

The São Paulo Approach Control (APP-SP) is responsible for an area of approximately 260 km and responsible for the movement of 320,179 aircraft in 2015, which represents 17.6% of the annual aircraft movement at airports administered by the Brazilian Company of Airport Infrastructure.^13^

The ATCs who work at this control area therefore tend to be at a higher risk for dysphonia because in addition to the natural stress of their work, they are subject to greater demands being placed on their voices than those experienced by ATCs from other operating bodies.^11^

We recently used a voice self-evaluation survey, designed by the Brazilian Ministry of Labor for teachers,^14^ to obtain an epidemiological profile of voice complaints (hoarseness and vocal tract discomfort) and risk factors in university teachers.^6^, ^7^, ^8^ University teachers show a high percentage of hoarseness and vocal tract discomfort (39.6% and 50.8%, respectively). Factors such as time of teaching, gender, work organization, workplace conditions, as well as personal habits and style/quality of life are related to these self-related symptoms.^7^, ^8^

In another recent study on the APP-SP, Villar et al.^11^ used a perceptual-auditory analysis of the /a/ vowel and observed that a high percentage of ATCs exhibited vocal alterations (44%), even among a group of subjects without vocal complaints.

We did not find any study about a self-evaluation survey in CTA. The use of this voice self-evaluation adapted to ATCs could provide information about the prevalence of hoarseness and vocal tract discomfort and related risk factors in this important group of professional voice users.

The aim of this study is to compare the self perceived hoarseness and vocal tract discomfort and risk factors in ATC in the APP-SP.

## Methods

This cross-sectional study was reviewed by the Ethics Committee in Research of the Federal University of São Paulo (CAAE 49357115.6.0000.5505) and was authorized by the Brazilian Aeronautics Command, from which the data were collected.

Voice self-evaluation forms, which were prepared by the Brazilian Ministry of Labor for teachers,^14^ and adapted to ATC, were completed by all 76 ATCs belonging to the APP-SP during a 1 month period in 2016.

The analyzed variables related to hoarseness and vocal tract discomfort were selected from the self-evaluation forms and grouped as follows:

Identification variables – age, gender, and time of work as an ATC (in years);

Work organization variables – professional activities other than ATC and professional activity that consumes the most time.

Workplace variables – noise, air pollution, air conditioning (the ATC chose between three options: comfortable, tolerable, and intolerable); and stress and anxiety due to the activity (three options: mild, moderate and very tense and stressful).

Voice care variables – care or medication for the throat or voice, seeking medical advice because of vocal symptoms, and the degree of difficulty of using the voice during work (none, mild, moderate, and severe).

Habits and style/quality of life outside the APP variables – use of voice, stress and anxiety (calm, tense and anxious, and very tense and anxious), drinking water/hydration, diet, smoking, alcohol consumption, coffee consumption, and health care (absent-minded, controlled/cautious, or concerned).

Differences in hoarseness and vocal tract discomfort rates for each variable were considered. Statistical analyses were performed using the SPSS statistical package for Windows, version 13.0. Student's *t*-test was used to compare hoarseness and vocal tract discomfort rates with the numerical variables; the chi-square test was used to compare hoarseness and vocal tract discomfort with the categorical variables; and the Fisher's exact or likelihood ratio test was used when necessary. A significance level of 5% (*p* < 0.05) was used.

## Results

The incidence of hoarseness and vocal tract discomfort in the sample of 76 ATCs was 19.7% and 38.2%, respectively. In total, 13.16% of ATCs presented both symptoms. The average and standard deviation time of those working as an ATC was 7.8 ± 6.6 years (between 1 and 37 years). The ATCs were 29.2 ± 6.5 years old and predominantly male (57.9%).

### Identification and work organization variables

No statistically significant differences were observed in the rate of hoarseness and vocal tract discomfort for identification and work organization variables (Table 1, Table 2, respectively).

**Table 1:** Comparison of identification variables as related to hoarseness and vocal tract discomfort.

Variables	Hoarseness		*p*-Value	Vocal tract discomfort		*p*-Value
	Yes	No		Yes	No	
*Time of working in years as an ATC*						
Mean (SD)	7.3 (3)	8 (7.3)	0.708^b^	8.2 (5)	7.6 (7.5)	0.741^b^
Median (Min.–Max.)	7 (3–14)	7 (1–37)		7 (2–25)	6 (1–37)	
Total	15	61		29	47	

*Age*						
Mean (SD)	28.5 (3.3)	29.4 (7.1)	0.626^b^	29.9 (6.5)	28.8 (6.6)	0.454^b^
Median (Min.–Max.)	29 (24–35)	27 (22–55)		28 (23–48)	27 (22–55)	
Total	15	61		29	47	

*Gender (percentage of population in parentheses)*						
Female	9 (60)	23 (37.7)	0.202^a^	13 (44.8)	19 (40.4)	0.890^a^
Male	6 (40)	38 (62.3)		16 (55.2)	28 (59.6)	
Total	15 (100)	61 (100)		29 (100)	47 (100)	

a Chi-square test.

b Student's *t* test.

**Table 2:** Comparison of work organization variables as related to hoarseness and vocal tract discomfort.

Variables	Hoarseness		*p*-Value	Vocal tract discomfort		*p*-Value
	Yes	No		Yes	No	
*Professional activities other than ATC*						
Yes	0 (0)	9 (14.8)	0.191^a^	2 (6.9)	7 (14.9)	0.469^a^
No	15 (100)	52 (85.2)		27 (93.1)	40 (85.1)	
Total	15 (100)	61 (100)		29 (100)	47 (100)	

*Professional activity that consumes the most time*						
ATC	0 (0)	6 (66.7)	Not calculated	2 (100)	4 (57.1)	0.500^a^
Other	0 (0)	3 (33.3)		0 (0)	3 (42.9)	
Total	0 (0)	9 (100)		2 (100)	7 (100)	

a Fisher's exact test. Percentage of population in parentheses.

### Workplace variables

In terms of air pollution, the percentage of vocal tract discomfort was higher among those who consider workplace intolerable than among those who considered their workplace comfortable and tolerable.

The percentage of vocal tract discomfort was also higher among those who considered their workplace to be a very tense and stressful environment than among those who considered their workplace to be a mild or moderately tense and stressful environment.

No statistically significant differences were observed in the rate of hoarseness and vocal tract discomfort for the other variables (Table 3).

**Table 3:** Comparison of workplace variables as related to hoarseness and vocal tract discomfort.

Variables	Hoarseness		*p*-Value	Vocal tract discomfort		*p*-Value
	Yes	No		Yes	No	
*Workplace in terms of noise*						
1. Comfortable	0	2		0	2	
2. Tolerable	8 (53.3)	40 (67.8)	0.456^a^	15 (51.7)	33 (73.3)	0.099^a^
3. Intolerable	7 (46.7)	19 (32.2)		14 (48.3)	12 (26.7)	
Total	15 (100)	59 (100)		29 (100)	45 (100)	

*Workplace in terms of air conditioning*						
**1. Comfortable**	**1 (6.7)**	**10 (16.4)**	**0.425**^a^	**4 (13.8)**	**7 (14.9)**	**0.292**^a^
2. Tolerable	7 (46.7)	32 (52.5)		12 (41.4)	27 (57.4)	
3. Intolerable	7 (46.7)	19 (31.1)		13 (44.8)	13 (27.7)	
Total	15 (100)	61 (100)		29 (100)	47 (100)	

*Workplace in terms of air pollution*						
1. Comfortable	1 (6.7)	13 (21.3)	**0.042**^a^	2 (6.9)	12 (25.5)	**0.014**^a^
2. Tolerable	7 (46.7)	38 (62.3)		16 (55.2)	29 (61.7)	
3. Intolerable	7 (46.7)	10 (16.4)		11 (37.9)	6 (12.8)	
Total	15 (100)	61 (100)		29 (100)	47 (100)	

*Workplace in terms of stress and anxiety*						
**Mild tense and stressful**	**0**	**1**		**0**	**1**	
Moderate tense and stressful	7 (46.7)	24 (40.0)	0.860^a^	6 (20.7)	25 (54.3)	**0.008**^a^
Very tense and stressful	8 (53.3)	36 (60.0)		23 (79.3)	21 (45.7)	
Total	15 (100)	60 (100)		29 (100)	46 (100)	

The categories in yellow were excluded from the analysis because there were 3 or fewer air traffic controllers in the group.

a Chi-square test. Bold indicates significant *p*-values. Percentage of population in parentheses.

### Voice care variables

The percentage of hoarseness was higher among those who had sought medical advice because of hoarseness. The percentage of hoarseness was also higher among those who experienced moderate difficulty working because of vocal problems.

No statistically significant differences were observed in the rate of hoarseness and vocal tract discomfort for the other variables (Table 4).

**Table 4:** Comparison of voice care variables as related to hoarseness and vocal tract discomfort.

Variables	Hoarseness		*p*-Value	Vocal tract discomfort		*p*-Value
	Yes	No		Yes	No	
*Degree of difficulty in voice use during work*						
None	4 (26.7)	27 (44.3)	<**0.001**^a^	8 (27.6)	23 (48.9)	0.115^a^
Mild	3 (20)	30 (49.2)		14 (48.3)	19 (40.4)	
Moderate	8 (53.3)	4 (6.6)		7 (24.1)	5 (10.6)	
Total	15 (100)	61 (100)		29 (100)	47 (100)	

*Care or medication for the throat or the voice*						
Yes	0 (0)	9 (14.8)	0.191^a^	4 (13.8)	5 (10.6)	0.725^a^
No	15 (100)	52 (85.2)		25 (86.2)	42 (89.4)	
Total	15 (100)	61 (100)		29 (100)	47 (100)	

*Seeking medical advice because of vocal symptoms*						
Yes	4 (26.7)	3 (4.9)	**0.025**^a^	4 (13.8)	3 (6.4)	0.417^a^
No	11 (73.3)	58 (95.1)		25 (86.2)	44 (93.6)	
Total	15 (100)	61 (100)		29 (100)	47 (100)	

a Fisher's exact test. Bold indicates significant *p*-values. Percentage of population in parentheses.

### Habits and style/quality of life variables

The percentage of vocal tract discomfort was higher among those who were stressed and anxious or very tense and anxious than among those who were calm. Moreover, the percentage of vocal tract discomfort was higher among those who were worried about health care.

No statistically significant differences were observed in the rate of hoarseness and vocal tract discomfort for the other variables (Table 5).

**Table 5:** Comparison of habits and style/quality of life variables as related to hoarseness and vocal tract discomfort.

Variables	Hoarseness		*p*-Value	Vocal tract discomfort		*p*-Value
	Yes	No		Yes	No	
*In terms of use of voice inside and/or the outside workplace, you describe yourself as a person who*						
1. Speaks little (introvert)	3 (20)	9 (14.8)	0.806^b^	5 (17.2)	7 (14.9)	0.954^a^
2. Speaks moderately (communicative)	8 (53.3)	38 (62.3)		17 (58.6)	29 (61.7)	
3. Speaks a lot (chattering)	4 (26.7)	14 (23)		7 (24.1)	11 (23.4)	
Total	15 (100)	61 (100)		29 (100)	47 (100)	

*In terms of stress and anxiety, you qualify yourself as a person who is*						
1. Calm	4 (26.7)	31 (50.8)	0.118^a^	8 (27.6)	27 (57.4)	**0.032**^a^
2. Tense and anxious	7 (46.7)	24 (39.3)		15 (51.7)	16 (34)	
3. Very tense and anxious	4 (26.7)	6 (9.8)		6 (20.7)	4 (8.5)	
Total	15 (100)	61 (100)		29 (100)	47 (100)	

*In terms of drinking water/hydration, you qualify yourself as a person who*						
1. Drinks a few liquids (forgets or does not feel thirsty and urinates less than 3× daily)	2 (13.3)	12 (19.7)	0.448^b^	4 (13.8)	10 (21.3)	0.614^a^
2. Drinks moderately (1 to 2 L/day)	9 (60)	41 (67.2)		21 (72.4)	29 (61.7)	
3. Drinks a lot (> 2 L/day)	4 (26.7)	8 (13.1)		4 (13.8)	8 (17)	
Total	15 (100)	61 (100)		29 (100)	47 (100)	

*In terms of diet, you qualify yourself as a person who*						
1. Eats a little (eats <3 meals/day)	1 (6.7)	6 (9.8)	0.451^b^	3 (10.3)	4 (8.5)	0.965^b^
2. Eats moderately (eats 3 meals a day)	9 (60)	44 (72.1)		20 (69)	33 (70.2)	
3. Eats a lot (does not control gluttony and recognizes this as a problem)	5 (33.3)	11 (18)		6 (20.7)	10 (21.3)	
Total	15 (100)	61 (100)		29 (100)	47 (100)	

*Cigarettes (tobacco)*						
1. Yes	0 (0)	3 (4.9)	0.197^b^	2 (6.9)	1 (2.1)	0.518^b^
2. No	15 (100)	54 (88.5)		26 (89.7)	43 (91.5)	
3. Former smoker	0 (0)	4 (6.6)		1 (3.4)	3 (6.4)	
Total	15 (100)	61 (100)		29 (100)	47 (100)	

*Alcohol*						
1. Yes	4 (26.7)	22 (36.1)	0.701^a^	10 (34.5)	16 (34)	1.000^a^
2. No	11 (73.3)	39 (63.9)		19 (65.5)	31 (66)	
Total	15 (100)	61 (100)		29 (100)	47 (100)	

*Coffee*						
1. Yes	10 (66.7)	43 (70.5)	0.762^c^	21 (72.4)	32 (68.1)	0.887^a^
2. No	5 (33.3)	18 (29.5)		8 (27.6)	15 (31.9)	
Total	15 (100)	61 (100)		29 (100)	47 (100)	

*In terms of health care, you consider yourself as being*						
1. Absent minded	5 (33.3)	16 (26.2)	0.298^b^	9 (31)	12 (25.5)	**0.044**^a^
2. Controlled/cautious	6 (40)	37 (60.7)		12 (41.4)	31 (66)	
3. Concerned	4 (26.7)	8 (13.1)		8 (27.6)	4 (8.5)	
Total	15 (100)	61 (100)		29 (100)	47 (100)	

a Chi-square test.

b Likelihood ratio test.

c Fisher's exact test. Bold indicates significant *p*-values. Percentage of population in parentheses.

## Discussion

In their systematic review, Cantor Cutiva et al.^15^ found wide variation in the prevalence of voice disorders and suggested that this variation may be due to the use of generic terms such as ‘vocal complaints’ and ‘vocal symptoms’ to describe these disorders. Thus, we were interested in using the voice self-assessment survey reformulated by the Ministry of Labor of Brazil, which examines symptoms such as hoarseness and vocal tract discomfort, to obtain an epidemiological profile of vocal complaints and risk factors in an ATC setting. In the present study, the incidence of vocal tract discomfort was almost twice that of hoarseness, at 38.2% and 19.7%, respectively.

Compared to university teachers using almost the same voice self-evaluation, in ATCs the prevalence of hoarseness and vocal tract discomfort was lower (19.7% vs. 39.6% and 38.2% vs. 50.8%, respectively).^6^, ^7^

Effective communication is essential between the pilot and ATCs. Moreover, ATCs work in a profession in which any disturbance in the communication provided during flights may lead to misunderstandings and compromise important flight information.

A previous study reported the presence of dysphonia in 44% of ATCs who were not always aware of the presence of this vocal deviation. The work environment and presence of risk factors, in addition to high levels of stress and great vocal demand, are combinations that can lead to the occurrence of dysphonia. If the main reason to seek medical care is the awareness of a problem, it must be considered that ATCs may not understand the seriousness of the presence of dysphonia, which postpones the diagnosis and increases the risk of a misleading communication between ATCs and pilots.

In this study, no statistically significant differences were observed in the rate of hoarseness and vocal tract discomfort for the variables “time of working as an ATC”, “age”, “gender”, “professional activities other than ATC” and “professional activities that consumes the most time”. Approximately 15% of ATCs reported other professional activities, which is far less than that among a sample of university teachers from a private institution in São Paulo (Brazil).^6^

With regard to the workplace, the percentage of both symptoms was higher among ATCs who considered their air pollution intolerable. Many studies have observed correlations between vocal complaints and air quality in teachers^16^, ^17^, ^18^, ^19^; this was specifically noted in a pilot study with an inhalation challenge test.^20^ Furthermore, the percentage of vocal tract discomfort was higher among those who considered their workplace a very tense and stressful environment. Nerrière et al. found an association between psychological distress and voice issues.^21^ Indeed, it should be noted that stress is associated with a phonation pattern due to greater effort by the laryngeal muscles, which is usually associated with vocal tract constriction, and poor vocal fold vibration, which reduces the efficiency of voice production and projection. Vocal tract compensation can be frequent, contributing to voice production worsening.

Although there was no association between vocal symptoms and workplace in terms of noise, Ishikawa et al.^22^ observed that dysphonic speech is relatively harder do understand in the presence of background noise as compared with normal speech.

Among ATCs, a small percentage (14.8%) reported receiving care or taking medications for their throat and/or voice. Additionally, only 9.2% sought medical advice because of vocal symptoms. Moreover, the percentage of hoarseness was higher among those who experienced moderate difficulty working because of vocal problems, which may explain the higher percentage of hoarseness among those who seek medical advice because of hoarseness. As stated before in the studies with university teachers,^7^ it is interesting how specific vocal symptoms markedly influence whether individuals seek medical care.

In contrast, the percentage of vocal tract discomfort was higher among those who were stressed and anxious and among those worried about their health care.

These findings justify increasing the care and guidance given to ATCs, not only in the workplace but in any environment.

The limitations of this study include the fact that the sample came from a single APP; thus, these data cannot be generalized to ATC in other APPs around the country.

## Conclusion

Among ATCs, the percentage of vocal tract discomfort was almost twice that of hoarseness. Both symptoms were present in ATCs who considered their workplace as having an intolerably polluted environment. Vocal tract discomfort was related to tense and stressful environments, and hoarseness was related to difficulty in using the voice at work.

## Conflicts of interest

The authors declare no conflicts of interest.
